# Human granulocytic anaplasmosis in Poland: clinical déjà vu or a new chapter in tick-borne diseases?

**DOI:** 10.1016/j.ijregi.2026.100940

**Published:** 2026-07-06

**Authors:** Agnieszka Joanna Wasilewska-Chrzanowska, Dawid Wojciulik, Piotr Czupryna, Justyna Adamczuk, Justyna Dunaj-Małyszko, Aneta Iżycka-Herman, Anna Grzeszczuk, Joanna Oklińska, Ewelina Kruszewska, Joanna Zajkowska, Anna Moniuszko-Malinowska

**Affiliations:** Department of Infectious Diseases and Neuroinfection, Medical University of Bialystok, Poland

**Keywords:** Anaplasmosis, Co-infection, Tick bite, Transaminase

## Abstract

**Objectives:**

Tick-borne diseases represent an increasing public health concern worldwide, including human granulocytic anaplasmosis (HGA). HGA is gaining relevance in travel medicine due to its expanding geographic distribution. Its true incidence remains unknown, largely due to underreporting and diagnostic challenges. HGA manifests itself by nonspecific symptoms and may be misdiagnosed as another tick-borne disease. This study aimed to direct focus on clinical manifestations of this disease and to examine the role of co-infection with other tick-borne pathogens on its clinical manifestations and laboratory results. An additional aim was to compare the obtained results with previous analysis performed on 120 patients.

**Methods:**

A retrospective clinical evaluation was conducted for all patients based on data extracted from their medical records. Each patient was assessed by the same team of physicians with extensive expertise in tick-borne diseases. The clinical assessment included a detailed history focused on key epidemiological and exposure-related factors. Patients had laboratory tests performed and all of them also had polymerase chain reaction tests to confirm infection. Overall, 22 patients with anaplasmosis were qualified.

**Results:**

This study presents symptoms that can differentiate patients with HGA mono-infection and co-infected group with significantly elevated aspartate aminotransferase observed in the mono-infection group.

**Conclusion:**

Including anaplasmosis in the differential diagnosis is essential in travel medicine, particularly in febrile patients with cytopenias following travel to endemic or rural areas. A thorough diagnostic approach—integrating epidemiological history, clinical manifestations (such as fever, cytopenias, transaminase elevation, especially elevated aspartate aminotransferase levels after excluding non-infectious causes), targeted laboratory investigations, and confirmatory polymerase chain reaction testing—is critical for timely and accurate identification of HGA.

## Highlights

• Assessment for *Anaplasma phagocytophilum* infection in cases with tick bite is mandatory.
• *A. phagocytophilum* infection frequently co-occurs with other tick-borne pathogens.
• Co-infections can significantly alter the clinical presentation.
• Human granulocytic anaplasmosis identification requires epidemiological history, laboratory, and polymerase chain reaction testing.

## Introduction

Tick-borne diseases represent an increasing public health concern worldwide. Ticks, belonging to various families and species, are distributed worldwide. They are essential vectors of infectious diseases, transmitting a broad spectrum of pathogens, including bacteria, viruses, and protozoa, which cause disease in humans and animals. The most widespread tick species in Europe is *Ixodes ricinus*, while *Ixodes scapularis* predominates in North America [1,2]. The occurrence of *Ixodes ricinus* in Europe has increased, and it occurs in Central European countries, such as Poland, Austria, the Czech Republic, Germany, Hungary, Slovakia, and Switzerland, and in Northern countries, such as Sweden or Norway [1,3,4]. Tick bites can lead to several diseases, including human granulocytic anaplasmosis (HGA) [5,6], which was reported for the first time in 1994 [7]. HGA is caused by the obligate Gram-negative intracellular bacteria, *Anaplasma phagocytophilum*. Horizontal transmission occurs by bite between ticks and vertebrate hosts, including humans, which predominates. Pathogens may be transmitted transstadially, involving nymphs and adult ticks, but transovarial transmission has not been described [8]. Anaplasmosis occurs in both genders and at any age; and is more frequent in men aged >40 years. Men have a higher risk of infection—the risk is 1.4 times higher compared with women [9]. Disease is found worldwide: in the USA, northern Europe, and south-eastern Asia [5]. According to the United States (US) Centers for Disease Control and Prevention reports*, A. phagocytophilum* infections are increasing. In 2000, there were 273 cases reported, 1010 cases were reported in 2008, 5762 in 2017, and 6729 in 2021 [10]. In Europe, human cases are not frequently reported [5]. The real epidemiology of HGA in Poland remains unknown, largely due to underreporting and diagnostic challenges, which increases the likelihood of missed cases among returning travelers. Symptoms of anaplasmosis are nonspecific and may overlap with other tick-borne diseases; thus, proper differential diagnosis is essential.

The current study aimed to analyze the variability of clinical manifestations in patients with anaplasmosis and to examine the role of co-infection with other tick-borne pathogens. An additional aim was to compare the obtained results with previous analysis performed on 120 patients.

## Methods

### *Study area*

The study was conducted in the Białystok county (coordinates 53.10, 23.19) in the north-eastern part of Podlaskie voivodeship in Poland. The region’s land use structure is characterized by a significant proportion of green space, with forests accounting for 29.2% and grassland 19.8% [11]. Patients were residents of Białystok, Sowlany, Olmonty, Ełk, Zaścianki, Fasty, Dzikie, Gródek, Drągi, and Grodziszcz.

### *Study design*

A retrospective clinical evaluation was conducted for all patients based on data extracted from their medical records. Each patient was assessed by the same team of physicians with extensive expertise in tick-borne diseases, ensuring diagnostic consistency and accuracy. The clinical assessment included a detailed history focused on key epidemiological and exposure-related factors, such as the timing and geographic location of the most recent tick bite, anatomical site and estimated duration of tick attachment, type of outdoor activity, place of residence, occupational exposure, prior history of tick-borne infections, status of tick-borne encephalitis (TBE) vaccination, and reported symptoms.

Patients were evaluated by the Department of Infectious Diseases and Neuroinfections and the outpatient department of the Medical University of Bialystok in 2022-2024 after exposure to a tick bite with various complaints, such as headache, vertigo, muscle pain, joint pain, fever, nausea and vomiting, and cranial and peripheral nerve paresis. Five (22.7%) patients were evaluated in the outpatient department and 17 (77.3%) were hospitalized. Inclusion criteria were: a tick bite or a possibility of a tick bite, symptoms of tick-borne disease, and a positive polymerase chain reaction (PCR) for *A. phagocytophilum*. The exclusion criterion was age under 18 years. Anaplasmosis was diagnosed according to the case definition by the Centers for Disease Control and Prevention when all three criteria were fulfilled (https://ndc.services.cdc.gov/case-definitions/ehrlichiosis-and-anaplasmosis-2008/).

(1) Clinical presentation: A tick-borne illness characterized by acute onset of fever and greater than or equal to one of the following symptoms or signs: headache, myalgia, malaise, anemia, leukopenia, thrombocytopenia, or elevated hepatic transaminases.
(2) Exposure: History of having been in potential tick habitat in the 14 days prior to the onset of illness or history of tick bite or history of tick bite.
(3) Laboratory criteria for diagnosis: Detection of *A. phagocytophilum* DNA in a clinical specimen via amplification of a specific target by PCR assay.

### *Blood tests*

All patients’ blood samples were examined by PCR for *A. phagocytophilum*.

*Methods-****DNA extraction.*** DNA was extracted with the Qiagen DNAeasy Blood and Tissue Mini kit (Qiagen Germany). The 200 μL K3-ethylenediaminetetraacetic acid whole blood (tube volume: 1.4 mL) was gently mixed and skin biopsies of 2 × 2 mm diameter were enzymatically processed at 56°C with proteinase K before extraction. DNA extraction was done on spin-columns according to the manufacturer’s guidelines and frozen at 20°C until used.

### *Molecular analysis for Anaplasma phagocytophilum DNA*

Amplification of *A. phagocytophilum* genetic material was performed with the diagnostic kit PCR Anaplasma (Blirt- DNA Gdańsk, Poland) coding a fragment of 16S rDNA gene encoding small ribosomal 16S RNA subunit. A conventional nested end-point PCR was performed with two pairs of primers (Sigma-Aldrich, Germany), for first amplification: gro607- F1 sens 5′ - GAA GAT GCT GTD GGA TGT ACT GC - 3′ and gro1294 -R1 antisens 5′ - AGT GCT TCA CCT TCT ACA TCT TC- 3′, for second amplification: gro667-F2 sens5′ - AAT ACT CAG AGT GCT TCT CAG TG- 3′ and gro1121-R2 antisens 5′- TGC ATR CCR TCA GTT TTT TCA AC-3′. Analyses were conducted in accordance with the manufacturers instruction, in the period from 2009 to 2011 in single PCR and in 2012 in a nested type of PCR. In conventional, single-course PCR in 2011, 1 μL of the template DNA isolate was added to 43.7 μL of the MasterMix with 5 μL of dNTPs and 0.3 μL of Hypernova polymerase for a final reaction mix volume of 50 μL. Amplification was performed in the following PCR program: initial denaturation at 94°C for 5 minutes, 35 cycles (denaturation at 94°C for 30 seconds, annealing at 56°C for 30 seconds, extension at 72°C for 3 seconds) and final extension at 72°C for 2 minutes. Positive results were 227 bp long fragments of the 16S rDNA gene. Nested PCR for *A. phagocytophilum* DNA detection was performed in two amplifications. In the first, PCR-OUT 2 μL of the template DNA isolates was added to 42 μL of the MasterMix with 5 μL of dNTPs and 1 μL of Taq nova polymerase for a final reaction mix volume of 50 μL. First amplification was performed in the following PCR program: initial denaturation at 95°C for 2 minutes, 40 cycles (denaturation at 94°C for 30 seconds, annealing at 55°C for 30 seconds, extension at 72°C for 60 seconds) and final extension at 72°C for 5 minutes. In a second amplification, PCR-IN, despite DNA isolate to 42 μL of the MasterMix with 5 μL of dNTPs and 1 μL of Taq nova, 2 μL of PCR product from first reaction was added. The PCR-IN program follows as in PCR-OUT, but in 30 cycles. Presence of the 16S rDNA gene fragments: 932 bp long in PCR-OUT and 546 bp long in PCR-IN attest to *A. phagocytophilum* infection. Lack of 932 bp long fragments in PCR-OUT does not exclude a positive result [12].

### *Molecular analysis for Borrelia species DNA*

Borrelia species molecular detection was performed by using the Borrelia burgdorferi PCR kit (GeneProof, Czech Republic) for in vitro diagnostics. The kit is designed for professional use in specialized clinical and research laboratories. The kit is designed for the detection of Borrelia burgdorferi sensu lato sp. group on the principle of amplification of the specific DNA sequence of a 276 bp fragment of flagellin encoding gene by nested one-tube PCR. The template DNA extract was added to 36 μL of the MasterMix for a final reaction mix volume of 40 μL. “Hot start” technology was used in the detection kit, minimizing risk of nonspecific reactions and maximizing sensitivity of procedure. Eventual PCR inhibition was controlled by internal standard in the reaction mix. Addition of uracil-DNA-glycosylase eliminated possible contamination during preparation of the reaction. Nested PCR was performed according to GeneProof instructions with our own modifications. The course of the amplification was prepared according to the following reaction program: Uracil-DNA Glycosylase decontamination at 37°C for 2 minutes, initial denaturation at 96°C for 10 minutes, first amplification for 30 cycles (denaturation at 96°C for 20 seconds, annealing at 68°C for 20 seconds, extension at 72°C for 40 seconds), second amplification for 45 cycles (denaturation at 96°C for 20 seconds, annealing at 54°C for 20 seconds, extension at 72°C for 30 seconds) and final extension at 72°C for 2 minutes. [12].

### *Immunoserology diagnostic*

The detection of TBE virus infection was performed with Euroimmun Anti-TBE Virus Enzyme-Linked Immunosorbent Assay (Euroimmun, Lübeck, Germany; TBE virus [TBEV] strain K23) diagnostic kit twice in patients with meningitis or encephalitis, that is, at the time of admission to the hospital and 2 weeks later. Anti/TBEV antibody level dynamics were observed. A level of viral specific immunoglobulin (Ig) M and IgG antibodies was marked according to manufacturer’s recommendations [13].

Co-infection was diagnosed if one patient was infected with at least two different pathogens. Co-infection with TBEV was stated if simultaneous infection with *A. phagocytophilum* and TBE was diagnosed, based on clinical symptoms, positive serology and lymphocytic pleocytosis in the cerebrospinal fluid. A case of TBE was diagnosed according to European Academy of Neurology guidelines [14].

Anaplasmosis co-infection with *B. burgdorferi* s.l. was defined on the basis of clinical presentation of erythema migrans (EM) or fulfilled criteria for neuroborreliosis. Lyme borreliosis was diagnosed according to Stanek et al. [15]. Neuroborreliosis was diagnosed according to European criteria [16].

### *Statistical analysis*

Statistical analysis was performed using Statistica 13.1. Different features observed in groups were compared by Mann-Whitney U and chi-square tests. *P*-value *<*0.05 was considered statistically significant.

## Results

Analysis of 22 patients (nine women [40.9%] and 13 men [59.1%]) at a mean age of 45.5 ± 15.1 (22-88) years with anaplasmosis was performed. Only two patients (9.0%) were older than 65 years old. Nine patients were city inhabitants (n = 40.9%) and 13 were village inhabitants (n = 59.1%). Less than half of the patients (n = 36.36%) remembered a tick bite. Time since a tick bite was 21.0 ± 7.7 days. In patients who presented with EM rash, the diameter ranged from 15 to 40 cm, with a mean of 23.3 ± 11.8 cm.

Co-infection was diagnosed if one patient was infected with at least two pathogens. Among all patients with *A. phagocytophilum* infection (22; 100%), six (27.3%) were co-infected with TBEV, and in eight patients (36.4%), co-infection with *B. burgdorferi* was found. Only one patient presented co-infection with both TBEV and Lyme disease (LD). Among all participants, EM was diagnosed in five cases. Eight patients presented neuroinfection symptoms, and seven of them had lumbar puncture performed. One of these patients was excluded from lumbar puncture due to pain reported by the patient during puncture attempt. In the group of eight patients with neuroinfection symptoms, mean age was 49.6 ± 10.3, and women accounted for 50% of the population. In the same group, five participants (62.5%) remembered the occurrence of a tick bite. Six participants (75%) were co-infected with TBEV, one participant (12.5%) had *Borrelia* co-infection, and one of them presented with both TBEV and *B. burgdorferi* co-infections (12.5%). The following numbers of patients in this group presented with meningeal signs: Brudziński’s sign: two (25%), Kerning’s sign: three (37.5%), Babiński’s sign: one (12.5%), Oppenheim’s sign: one (12.5%).

Table 1 presents the frequency of reporting given symptoms in each group of patients: successively with anaplasmosis, HGA and LD co-infection, HGA and TBE co-infection, HGA, TBE, and LD co-infection, and among all subjects with co-infections combined. According to Table 1, headache, nausea, and vomiting have been more often observed in cases with *A. phagocytophilum* mono-infection when compared with the co-infection group. The following symptoms, such as vertigo, muscle pain, joint pain, and fever, were more frequent in all co-infections than in cases of HGA mono-infection. Skin lesions resembling EM were not observed in any case of *A. phagocytophilum* mono-infection, neither were meningeal signs nor neck stiffness observed. Comparison of the HGA + TBE group with the HGA + LD group revealed that headache, vertigo, and fever were more frequent in the first-mentioned group than in the second one. Fever was more frequent in the HGA + TBE group than in the group without co-infection. Kernig’s or Brudziński’s sign was present only in the HGA + TBE group. One person with HGA + LD presented neck stiffness; however, this symptom was primarily observed in the HGA + TBE group (83.3%).

**Table 1:** Symptoms declared by patients with mono- and co-infection.

Symptoms	All patients (n = 22)	HGA (n = 7)	HGA+ LD (n = 8)	HGA +TBE (n = 6)	HGA + TBE + LD (n = 1)	All co-infections (n = 15)
Headache	12 (54.5%)	5 (71.4%)	1 (12.5%)	5 (83.3%)	1 (100.0%)	7 (46.7%)
Vertigo	4 (18.2%)	1 (14.3%)	1 (12.5%)	2 (33.3%)	0 (0.0%)	3 (20.0%)
Nausea	2 (9.1%)	2 (28.6%)	0 (0.0%)	0 (0.0%)	0 (0.0%)	0 (0.0%)
Vomiting	3 (13.6%)	2 (28.6%)	0 (0.0%)	0 (0.0%)	1 (100.0%)	1 (6.7%)
Muscle pain	2 (9.1%)	0 (0.0%)	2 (25.0%)	0 (0.0%)	0 (0.0%)	2 (13.3%)
Joint pain	4 (18.2%)	1 (14.3%)	3 (37.5%)	0 (0.0%)	0 (0.0%)	3 (20.0%)
Fever	8 (36.4%)	1 (14.3%)	1 (12.5%)	5 (83.3%)	1 (100.0%)	7 (46.7%)
Skin lesion resembling EM	5 (22.7%)	0 (0%)	5 (62.5%)	0 (0.0%)	NN	5 (33.3%)
Kernig’s or Brudziński’s sign	3 (13.6%)	0 (0.0%)	0 (0.0%)	3 (50.0%)	NN	3 (20.0%)
Neck stiffness	7 (31.8%)	0 (0.0%)	1 (12.5%)	5 (83.3%)	1 (100.0%)	7 (46.7%)

Abbreviations: EM, erythema migrans; HGA, human granulocytic anaplasmosis; LD, Lyme disease; NN, not noted; TBE, tick-borne encephalitis.

Table 2 presents the results of basic laboratory parameters measured in patients with *A. phagocytophilum* mono-infection and co-infections. Analysis revealed a statistically significant difference in aspartate aminotransferase (AST) levels between the mono-infection group and the combined co-infection group (*P* = 0.01), with elevated AST observed in the mono-infection group (60.17 vs 18.27 U/l). Differences in mean C-reactive protein concentrations were also noted, with higher levels in the HGA group. Additionally, alanine aminotransferase levels were also increased in patients with mono-infection; however, these differences were not statistically significant.

**Table 2:** Results of basic laboratory tests in *A. phagocytophilum* mono-infections and co-infections.

	*A. phagocytophilum mono-infection*					*A. phagocytophilum co-infections*					*P*-value
	Min	Max	Mean	SD	Me	Min	Max	Mean	SD	Me	
WBC (tys/μL)	2.73	9.15	6.44	2.31	6.58	4.82	13.99	8.57	3.00	8.20	0.250
RBC (mln/μL)	4.07	5.81	4.84	0.57	4.80	3.85	5.62	4.74	0.59	4.67	0.820
HGB (g/dL)	11.5	17.3	14.56	2.06	14.80	8	17	13.80	2.49	14	0.616
HCT(%)	34.8	50.3	41.72	5.14	40.90	27.10	47.70	40.21	5.94	40.85	0.820
PLT (tys/μL)	98	270	187.33	63.60	197.00	153	359	236.75	57.42	230	0.180
CRP (mg/l)	0.6	259	44.65	95.87	1.47	0.6	34.80	5.08	10.61	1.24	0.635
Glucose (mg/dL)	72	133	100.67	23.50	99.00	73	141	96.75	20.83	94	0.950
Creatinine (mg/dL)	0.73	1.11	0.91	0.14	0.91	0.59	1.07	0.90	0.16	0.95	0.808
Fibrinogen (mg/dL)	141	625	353.67	175.87	351.50	200	390	315.80	61.56	315.50	0.792
Bilirubin (mg/dL)	0.65	1.23	0.94	0.29	0.93	NN	NN	NN	NN	NN	NN
AST (U/l)	19	232	60.17	84.35	28.00	10	32	18.27	6.72	16.00	**0.0103**
ALT (U/l)	11	123	44.33	44.78	23.50	9	36	19.73	7.90	18	0.591
LDH (U/l)	168	263	215.50	67.18	215.50	NN	NN	NN	NN	NN	NN
CK (U/l)	370	370	370	NN	370	NN	NN	NN	NN	NN	NN

Abbreviations: ALT, alanine aminotransferase; AST, aspartate aminotransferase; CK, creatine kinase; CRP, C-reactive protein; HCT, hematocrit; HGB, hemoglobin; LDH, lactate dehydrogenase; Me, median; NN, not noted; PLT, platelet count; RBC, red blood cell count; WBC, white blood cell count.

The boldface indicates a statistically significant difference in aspartate aminotransferase (AST) levels between the monoinfection group and the combined co-infection group (*P* = 0.01), with elevated AST observed in the mono-infection group (60.17 vs 18.27 U/l).

Table 3 illustrates that the duration of hospitalization among patients with combined co-infections was relatively longer than among patients with mono-infection. However, this difference in length of hospitalization, averaging 12.64 days in the co-infection group vs 9.17 days in the mono-infection group, was not statistically significant.

**Table 3:** Duration of hospitalization.

	*A. phagocytophilum* mono-infection					*A. phagocytophilum* co-infections					*P*-value
	Min	Max	Mean	SD	Me	Min	Max	Mean	SD	Me	
Hospitalization time (days)	3	17	9.17	5.40	7	6	30	12.64	6.30	10	0.680

Abbreviations: Max: maximum; Me: median; Min: minimum.

## Discussion

The true incidence of HGA remains unknown, largely due to underreporting and diagnostic challenges, which increases the likelihood of missed cases among returning travelers. Long-distance transport of ticks by travelers represents an underrecognized pathway for the global spread of ticks and tick-borne pathogens [17]. Human-mediated travel poses the risk for the increase in the number of HGA cases in the world and the risk of potential introduction of non-native arthropod species and pathogens, which additionally hinders proper diagnostics of tick-borne diseases. With billions of airline passengers and extensive global travel by land and sea each year [18], the hidden risk of humans transporting ticks is likely far greater than currently recognized, posing a silent but significant threat to public health worldwide [17]. Additionally, HGA is gaining relevance in travel medicine due to its global expansion driven in part by climate change. Increased temperature increases the survival and activity period of ticks, increases the range of both reservoir and tick hosts (e.g. mice and deer), and increases the duration of the season when people may be exposed to ticks [19]. Including anaplasmosis in the differential diagnosis is essential in travel medicine, particularly in febrile patients with cytopenias following travel to endemic or rural areas.

HGA manifests with diverse clinical symptoms, although it may occasionally remain asymptomatic. The predominant clinical features include high-grade fever, intense headache, myalgia, and general malaise. Additionally, patients may present with less frequent symptoms such as arthralgia, weight loss, cough, and nausea. A minority of patients will develop life-threatening complications [[20], [21], [22]]. Patients in the current study presented with mild symptoms, and no severe outcomes have been noted. Abnormalities in laboratory tests described in the literature include: thrombocytopenia, leukopenia, and elevated transaminase levels [23].

All mentioned symptoms are nonspecific and may wrongly suggest other tick-borne diseases, such as LD or TBE. Thus, only prompting differential diagnosis based on clinical signs and laboratory tests may be problematic. Proper diagnosis of anaplasmosis includes clinical signs, patient’s history, laboratory tests, serology, blood smear, and PCR [5]. Additionally, knowing that *Ixodes ricinus* can also be a vector for LD and TBE, distinguishing these infections promptly can also be challenging.

In 2014, Welc-Falęciak et al. [24] described the prevalence of rarer tick-borne pathogens such as *Rickettsia, Anaplasma, Ehrlichia,* and *Candidatus Neoehrlichia* spp*.* in *Ixodes ricinus,* collected from natural and urban area in central and north-eastern Poland. Among 1325 collected ticks—82 (6.2%) were infected with at least one pathogen. Rickettsia spp. was detected in 58 ticks (4.4%), *A. phagocytophilum* in 22 ticks (1.7%) whereas *Ehrlichia* spp. and *Candidatus Neoehrlichia* spp*.* were identified in four ticks (0.3%) and three ticks (0.2%), respectively.

In 2022, Grochowska et al. [25] described the occurrence of tick-borne pathogens in ticks collected from natural and urban area in north-eastern Poland. The analyzed material comprised 842 *Ixodes ricinus* and 270 *Dermacentor reticulatus.* Of the 1112 examined ticks, 249 (22.4%) tested positive for at least one pathogen. Among *I. ricinus, Borrelia* spp. was detected in 212 specimens (25.2%), *Babesia* spp, in 17 ticks (2.0%) and *A. phagocytophilum* in 10 ticks (1.2%). In *D. reticulatus, Babesia* spp. was the most frequently identified pathogen detected in 19 ticks (7.0%), followed by *A. phagocytophilum* in three ticks (1.1%) and *Borrelia* spp. in two ticks (0.7%). The presence of *Rickettsia* spp*., Bartonella* spp. or *Coxiella burnetii* was not detected [25].

We attempted to compare the symptoms presented by the group with *A. phagocytophilum* mono-infection and *A. phagocytophilum* co-infections. Interestingly, in our study, only 32% of all patients with anaplasmosis presented no co-infections; the rest were either positive for LD (36%), TBE (27%), or both LD and TBE (4.5%). The high co-infection rate of patients with *A. phagocytophilum* infection in tick-endemic areas was previously described by Moniuszko-Malinowska et al. [5], which aligns with the results obtained in the current study. These observations result from the co-existence of more than one pathogen in one vector, or the effect of multiple bites by different ticks [26]. Additionally, it has been proven that individuals seropositive for Lyme borreliosis are more likely to have anti-HGA antibodies than seronegative ones [27]. Grzeszczuk et al. [28] described two confirmed acute HGA cases (fulfilling the European case definition criteria defined by European Society of Clinical Microbiology and Infectious Diseases Study Group on Coxiella, Anaplasma, Rickettsia, and Bartonella (ESCAR)) for the first time in a prospective manner in Poland. They evaluated 68 patients who had fever within 4 weeks after a tick bite, four of which was HGA positive (in one patient by PCR, in another by seroconversion, in two further individuals by immunofluorescence). Both confirmed HGA cases and seropositive individuals had *A. phagocytophilum* infection, concurrent with Lyme borreliosis or TBE.

According to a meta-analysis of clinical manifestations and laboratory abnormalities in patients with HGA performed by Dumler et al. [6], frequent laboratory abnormalities found in *A. phagocytophilum* infection are: thrombocytopenia, leukopenia, anemia, and elevated hepatic transaminase levels. Our findings revealed elevated AST levels in the HGA mono-infection group compared with the group with co-infections, and the results were statistically significant. Our findings are in agreement with previous observations presented in our previous study, where we compared anaplasmosis mono-infection and co-infection with either LD, TBE, or both. Differences in C-reactive protein concentration and AST activity (higher in patients with co-infection) were observed. Additionally, we have previously observed a difference in platelet count, but at the border of statistical significance [5]. In another study, examination of the Indian population performed by Vinayaraj et al. [29] revealed that elevated liver enzymes were more frequent in HGA infection than in the HGA-negative population.

According to our observations, specific symptoms tend to overlap; for example, headache was present in 71.4% of patients with *A. phagocytophilum* mono-infection, and 83.3% with HGA+TBE co-infection. We also observed a tendency of several symptoms to potentiate: fever was present in 14.3% of patients with HGA mono-infection, and in the HGA + TBE co-infection group, this symptom was present in a higher percentage of participants (83.3%). These results suggest possible interactions between the analyzed pathogens. The rate of fever among participants with HGA was higher than in the HGA + LD group; however, these results were not statistically significant. Surprisingly, none of our patients in the mono-infection group presented with muscle pain.

In our study, patients with anaplasmosis were treated with doxycycline—the drug of choice for adults with *A. phagocytophilum* infection—and all presented full recovery; this observation proves the positive therapeutic effect of the currently recommended treatment.

## Conclusions

• Clinicians must maintain a high index of suspicion for *A. phagocytophilum* infection in any patient with a recent tick bite, regardless of initial symptom severity. Failure to consider this diagnosis may lead to delayed treatment and complications.
• *A. phagocytophilum* infection frequently co-occurs with other tick-borne pathogens, most notably TBEV and *Borrelia burgdorferi*, underscoring the need for comprehensive diagnostic screening in endemic areas.
• Co-infections involving *A. phagocytophilum* and either TBEV or *B. burgdorferi* can significantly alter the clinical presentation, potentially masking or exacerbating individual symptoms, and complicating diagnosis and management.
• A thorough diagnostic approach—integrating epidemiological history, clinical manifestations (such as fever, cytopenias, transaminase elevation), targeted laboratory investigations, and confirmatory PCR testing—is critical for timely and accurate identification of HGA.
• All patients responded favorably to doxycycline treatment, achieving full clinical recovery, which reinforces the efficacy of current therapeutic protocols and highlights the importance of prompt empirical therapy in suspected cases.
• Including anaplasmosis in the differential diagnosis is essential in travel medicine, particularly in febrile patients with cytopenias following travel to endemic or rural areas.

## CRediT authorship contribution statement

**Agnieszka Joanna Wasilewska-Chrzanowska:** Writing – review & editing, Writing – original draft, Visualization, Investigation, Data curation. **Dawid Wojciulik:** Writing – review & editing, Writing – original draft, Visualization, Investigation, Data curation. **Piotr Czupryna:** Software, Methodology, Investigation, Data curation. **Justyna Adamczuk:** Visualization, Investigation, Data curation. **Justyna Dunaj-Małyszko:** Visualization, Methodology, Data curation. **Aneta Iżycka-Herman:** Visualization, Methodology, Data curation. **Anna Grzeszczuk:** Visualization, Investigation, Data curation. **Joanna Oklińska:** Visualization, Investigation, Data curation. **Ewelina Kruszewska:** Visualization, Methodology, Data curation. **Joanna Zajkowska:** Visualization. **Anna Moniuszko-Malinowska:** Writing – review & editing, Validation, Resources, Project administration, Funding acquisition, Conceptualization.

## Declaration of competing interest

The authors declare that they have no known competing financial interests or personal relationships that could have appeared to influence the work reported in this paper.
